# Comparison between real-world practice and application of the FRAX algorithm in the treatment of osteoporosis

**DOI:** 10.1007/s40520-022-02212-x

**Published:** 2022-08-16

**Authors:** Francesca Zoccarato, Chiara Ceolin, Caterina Trevisan, Anna Citron, Labjona Haxhiaj, Aurelio Guarnaccia, Matteo Panozzo, Carlotta Campodall’Orto, Alessandra Coin, Sandro Giannini, Giuseppe Sergi

**Affiliations:** 1 Department of Medicine (DIMED), Geriatrics Division, University of Padua, Via Giustiniani 2, 35128 Padua, Italy; 2 Department of Medicine (DIMED), Clinica Medica I, University of Padua, Padua, Italy

**Keywords:** Osteoporosis, Older persons, FRAX, Multidimensional approach

## Abstract

**Background and aims:**

The most recent guidelines suggest treating patients whose FRAX 10-year fracture risk scores are ≥ 20%. However, this method of evaluation does not take into account parameters that are nonetheless relevant to the therapeutic choice. Our aim was to compare the therapeutic choices for treatment based on a wider assessment (real-world practice) with those based on FRAX scores, taking 20% as the cut-off score.

**Methods:**

We obtained the medical history, bone mineral density (BMD) values, and the presence of major fragility fractures in a sample of 856 postmenopausal women. The 10-year FRAX risk of major osteoporotic fracture was calculated, and patients were grouped into risk classes (“FRAX < 20%” = low, “FRAX ≥ 20%” = high); we then compared the treated and untreated patients in each class. After an average interval of 2.5 years, changes in lumbar and femoral BMD and appearances of new fragility fractures were recorded.

**Results:**

83% of high-risk patients and 57% of low-risk patients were treated. The therapeutic decision was based mainly on densitometric values and the presence of vertebral fractures. At the 2.5 year follow-up, lumbar spine and femur BMD had decreased in the untreated group; 9.9% of the treated patients developed new vertebral fragility fractures, compared with 5.3% of the untreated patients.

**Discussion and conclusions:**

Our wider assessment designated as at high fracture risk a group of patients who had not been identified by the FRAX assessment. FRAX could underestimate the risk of fracture in older people, for which the therapeutic choice should consider a broader approach, also based on individual patient’s needs.

## Introduction

Osteoporosis is a pathology of the skeletal tissue characterized by diminished bone strength related to a reduction in bone mineral density and qualitative changes in the micro and macro architecture [1]. The complication of greatest concern is fracture. Osteoporotic fractures are associated with increased morbidity, as they cause a large number of physical and psychological problems, including pain, depression, subsequent fractures, impaired function, and disability [2]. For this reason, the most recent guidelines recommend treatment aimed at preventing fragility fractures in subjects at high risk of fracture [3]. Bone mineral density (BMD) does not provide very accurate risk estimates because more than half of fragility fractures occur in non-osteoporotic patients [4]; although *T* scores < − 2.5 SD indicate a high risk of fracture at younger ages, the association progressively decreases with advancing age [5–8].

Major fracture risk factors must be taken into account when deciding on treatment, especially with the onset of old age. With this in mind, in 2008 the University of Sheffield launched the Fracture Risk Assessment Tool, known as FRAX [2]. The tool is a simple and easy to use means of calculating fracture risk in both men and women using an algorithm based on easily identifiable risk factors taken mainly from the patient’s history (previous fragility fracture, parental hip fracture, smoking, systemic use of glucocorticoids, excessive alcohol intake, rheumatoid arthritis, and other causes of secondary osteoporosis), in addition to age and gender, and body mass index (BMI), to estimate the 10-year probability of fracture; when available, the BMD value (*T* score) of the femur can also be entered in the FRAX [9]. The National Osteoporosis Foundation recommends treating patients whose FRAX 10-year risk scores are ≥ 20% for major osteoporotic fractures and ≥ 3% for hip fracture to reduce their risk of fracture [10, 11].

However, in clinical practice physicians often take an empirical approach to therapy, and consider factors other than FRAX scores. In fact, in treating the older persons, factor such as the patient’s compliance with treatment, polypharmacy, and the risk/benefit ratio for the individual have considered. It is, therefore, important that the decision to start treatment for osteoporosis be based on a wider assessment rather than the mere application of a mathematical algorithm.

These considerations lead us to expect that this approach to therapy decision making in the real-world will improve results in terms of patient selection and treatment efficacy. Our aim was to investigate what could be the main factors influencing the specialist clinical decision in the real-world, and to assess how consensual our clinical decision was to FRAX in predicting fracture risk.

## Materials and methods

### Study population

This retrospective study was conducted on 856 patients at the outpatient clinic for the diagnosis and treatment of osteoporosis of the Geriatrics Division of the University of Padova. The period of recruitment was from January 2018 to December 2020. The inclusion criteria were: postmenopausal female patients; between 40 and 90 years of age; having undergone at least two outpatient clinical evaluations (first visit and re-evaluation after an average interval of 2.5 years).

The study was designed in accordance with the Helsinki Declaration and was authorized by the local Ethics Committee (Comitato Etico per la sperimentazione clinica di Padova, number 0031124). All participants were fully informed of the nature, purpose and procedures of the study, and gave their written informed consent.

### Data collection

#### Patient characteristics

The following physiological, clinical, and pharmacological data were collected for each participant during a medical interview by experienced physicians: age of menarche; months of breastfeeding; age and type of menopause (physiological, surgical or drug-induced); family history of osteoporotic fracture (familiarity); smoking habit (active or previous smoker, and relative duration, severity and exposure); consumption of alcohol and coffee; calcium dietary intake; physical activity; number of falls (a number of falls greater than two in a year is an expression of susceptibility to falling); anamnestic presence of endocrine-metabolic disorders (hypothyroidism or hyperthyroidism, hypogonadism, diabetes), gastrointestinal diseases (malabsorption syndromes, gastritis, esophagitis, chronic liver diseases), current or previous neoplasms, rheumatological and neuromuscular diseases (connective tissue diseases, rheumatoid arthritis), organ transplants; use of thyroid hormones, estrogen–progestins, cortisones, immunosuppressants, chemotherapy, anticoagulants, anticonvulsants, NSAIDs, benzodiazepines, calcium and vitamin D supplements, and calcium-sparing diuretics; presence of vertebral or femoral fragility fractures; any therapy prescribed at the first outpatient evaluation. Finally, patients were assessed for expectations, autonomy, family conditions, therapy management and adherence, the possibility of reducing risk factors, and individual cost–benefit ratio.

Each participant’s body weight and height were measured by a trained physician and body mass index (BMI) was calculated as the ratio of weight to height squared (kg/m^2^).

#### Biochemical data

The following biochemical parameters were analyzed in venous blood samples: serum calcium and phosphate, vitamin D and parathyroid hormone (PTH), bone alkaline phosphatase (ALP), thyroid-stimulating hormone (TSH), free thyroxine (FT4), alanine transaminase (ALT), aspartate transaminase (AST), creatinine.

From the 24 h urine collection we obtained the 24 h calcium and 24 h phosphate values. The analyses were performed following standard procedures at the laboratory unit of the University Hospital of Padua, which has Clinical Pathology Accreditation.

#### Radiographic examination

All patients underwent dual-energy X-ray absorptiometry (DXA) using fan-beam technology (Hologic QDR 4500 W: Hologic Inc.) to assess lumbar spine (L1–L4) and total femur BMD and *T* scores. Normal BMD is defined by a *T* score between + 2.5 and − 1.0 SD; osteopenia (low BMD) is defined at a *T* score between − 1.0 and − 2.5 SD; osteoporosis is defined by a *T* score lower than − 2.5 SD; overt osteoporosis is defined by a *T* score lower than − 2.5 SD and by the simultaneous presence of one or more fragility fractures.

Vertebral fractures were identified by lateral radiography of the thoracic and lumbar regions by qualified medical practitioners, specialists in the field of geriatrics and osteoporosis. Each scan was evaluated by two examiners who discussed any disagreements until consensus was reached. Using a protocol based on the anterior, middle, and posterior heights of each vertebra measured with the aid of a caliper, the presence of a vertebral fracture was diagnosed when there was ≥ 20% reduction in the anterior, middle or posterior vertebral height or when there was a loss in vertebral body height relative to an adjacent normal-looking vertebra, according to the criteria proposed by Genant [12]. Vertebral fractures were assessed after taking into account deformities linked to spinal curvatures (scoliosis, or an accentuated thoracic kyphosis or lumbar lordosis) with parallax distortion of the vertebral borders, osteoarthritis, degenerative disk disease or Schmorl’s nodes. Where available, sequential radiographs were evaluated and compared to confirm the presence of incident vertebral fractures. For the purposes of our study, we defined vertebral fractures as “unknown”, if they were detected for the first time from the radiography required at our outpatient clinic and if they had not previously been clinically or anamnestically reported, and “known” if they were reported by patients due to clinical symptoms or previous investigations, and then confirmed by our radiography.

#### Fracture risk assessment

The 10-year probability of fracture—in particular, of a major osteoporotic fracture and a hip fracture-for each patient was retrospectively calculated using the FRAX algorithm. The variables considered were: nationality, age, sex, weight, height, history of previous fragility fractures, history of fragility fractures in parents, active smoking, glucocorticoid treatment, rheumatoid arthritis, other causes of secondary osteoporosis, high consumption of alcohol, and bone density measured at the femoral neck.

#### Wider assessment

The decision to treat these patients considered the traditional risk factors for osteoporosis, but in the doctor’s decision also other factors were evaluated, such as patients’ ability to adhere to treatment, the possibility of correcting risk factors, patient’s willingness to be treated, family context, the cost–benefit ratio, and polypharmacy.

#### Follow-up visit

After the first visit, a follow-up evaluation was scheduled about 18 months after. In this occasion, anamnestic data were revised, incident major fractures were recorded, and each patient was asked to bring in vision a new densitometry, a recent dorsal-lumbar spine X-ray and blood chemistry tests of the phospho-calcium metabolism performed shortly before.

### Statistical analysis

The characteristics of the sample were expressed as means ± standard deviation (SD) for continuous quantitative variables, and as number and percentages for categorical variables. The patients were first categorized as either “treated”, i.e., subjects who were given bisphosphonates, Denosumab and Teriparatide after the first outpatient evaluation, or “untreated”, i.e., patients who did not undergo any therapy, or were only recommended calcium and vitamin D supplements and calcium-sparing diuretics to correct any underlying metabolic alterations and bone loss. Subsequently, patients were classified according to their FRAX scores as having a low or high risk of major osteoporotic fracture (FRAX < 20 and FRAX ≥ 20, respectively). The characteristics of the various groups were compared by Student’s *t* test or an analysis of variance (ANOVA) for quantitative variables, and by Pearson’s chi-squared test with Bonferroni’s correction for categorical variables. A decision tree was built to determine the factors that lead to the treatment choices observed in our study. We ran a decision tree analysis with a chi-squared automatic interaction detection (CHAID) algorithm [13, 14]. In particular, the variables showing a *p* value < 0.10 on multivariate logistic regression were included in the CHAID analyses. The decision tree consisted of a flowchart with nodes that split and formed branches. The CHAID algorithm is a non-parametric procedure and, therefore, it required no assumptions to be made of the underlying data. Multiple 2 × 2 contingency tables between the dependent variable and each independent variable were created; the most significant independent variable in a chi-squared test was then selected to branch out the decision tree. We set the maximum number of splits to four, the minimum number of cases in the parent node to 50, and the minimum number of cases in the child node to 20 to contain the number of branching points and preserve statistical power [15]. A *p* value < 0.05 in the chi-squared statistic, adopting Bonferroni’s correction, was considered significant for node splitting purposes in the decision tree analysis. Of the 856 individuals included in the present study, complete information on all variables included in the CHAID analysis was available for all patients. As the proportion of missing data for each variable was insignificant, multiple imputations were not used. The final model was evaluated by calculating the misclassification risk estimate and the overall accuracy percentage (which is the probability that an individual will be correctly classified by a test). A tenfold cross-validation of the decision tree was carried out to confirm the misclassification risk of the decision tree estimated for the sample as a whole. Misclassification risk refers to the misplacement of a patient in a specific group; for each tree, misclassification risk is estimated by applying the tree to the subsample excluded in generating it [15].

All analyses were performed in the Statistical Package for Social Science 21.0 software (SPSS, Armonk, NY: IBM Corp) with the significant level set at *p* < 0.05.

## Results

The characteristics of the sample at baseline, and the differences between the treated and untreated subjects are shown in (Table 1). The treated subjects (61.7% of the total) were older and had significantly lower densitometric values of the femur and spine. Physiological menopause was less frequent in treated women, and their rate of vertebral osteoporotic fractures was significantly higher (45.1 vs 13.7%, *p* < 0.001). The 10-year FRAX risk scores for major osteoporotic fracture were 15.0% for the treated patients vs 9.5% for untreated patients.

**Table 1:** Characteristics of the sample at baseline

	All	Treated	Untreated	*p value*
	(*n* = 856)	(*n* = 528)	(*n* = 328)	
Age [years]	63.7 ± 9.9	65.1 ± 9.7	61.3 ± 9.7	< *0.001*
BMI [kg/m^2^]	23.9 ± 4.0	23.8 ± 3.9	24.0 ± 4.2	0.26
Familiarity [%]	33.8	32.8	35.7	0.55
Age at menopause [years]	49.2 ± 4.9	49.1 ± 5.1	49.4 ± 4.7	0.37
Type of menopause [%]				
Early menopause	16.9	17.3	16.3	0.85 *0.025* 0.89 0.21
Physiological menopause	81.1	78.6	85.2	
Surgery menopause	15.7	17.1	13.4	
Pharmacological menopause	3.3	4.2	1.7	
Breastfeeding [months]	9.6 ± 11.0	9.9 ± 11.7	9.0 ± 9.6	0.87
Current smoking habits [%]	8.2	8.3	7.9	0.91
Previous smoking habits [%]	18.5	16.7	21.3	0.54
Years smoking	24.8 ± 14.3	25.7 ± 14.9	23.6 ± 13.4	0.49
Susceptibility to falling [%]	7.5	8.5	5.8	0.84
Diabetes [%]	3.7	3.6	4.0	0.23
Drugs [%]				
Oral glucocorticoids	10.6	12.7	7.3	*0.013*
Aromatase inhibitors	0.7	0.4	1.2	0.21
Anticoagulants	1.8	1.3	2.4	0.24
DEXA *T* score				
Lumbar spine	− 2.5 ± 1.09	− 2.7 ± 1.1	− 2.1 ± 1.0	< *0.001*
Total hip	− 1.7 ± 0.82	− 1.9 ± 0.8	− 1.5 ± 0.8	< *0.001*
Vertebral fractures [%]	33.1	45.1	13.7	< *0.001*
Femur fractures [%]	3.3	4.2	1.8	*0.062*
FRAX				
Major fractures	12.85 ± 9.92	15.0 ± 10.7	9.5 ± 7.3	< *0.001*

Statistically significant *p* values are in italics

Numbers are means ± SD, medians (interquartile range), or counts (%), as appropriate

Table 2 shows the patients grouped according to their FRAX profiles (using 20% as the cut-off in accordance with the National Osteoporosis Foundation’s guidelines). 83% of patients considered high risk (> 20%) were treated with anti-osteoporotic drugs. This group included patients with lower lumbar densitometric values and higher vertebral fracture rates. 57% of the subjects considered at low risk (< 20%) were also treated: these patients were older and took cortisone more frequently, had lower densitometric values of the lumbar site and femur (− 2.7 ± 1.0 vs − 2.1 ± 1.0, *p* < 0.001, and − 1.7 ± 0.8 vs − 1.4 ± 0.8, *p* < 0.001, respectively), had experienced more vertebral fractures (33.5 vs 11.6%, *p* < 0.001), and had a significantly higher FRAX 10 year risk.

**Table 2:** Characteristics of the sample grouped as high risk (FRAX ≥ 20) or low risk (FRAX < 20)

FRAX								
< 20					≥ 20			
Characteristics	All	Treated	Untreated	*p *value	All	Treated	Untreated	*p *value
	(*n* = 708)	(*n* = 406)	(*n* = 302)		(*n* = 148)	(*n* = 122)	(*n* = 26)	
Age [years]	61.7 ± 8.9	62.6 ± 8.6	60.4 ± 9.1	< *0.001*	73.4 ± 8.7	73.6 ± 8.5	72.4 ± 9.8	0.41 0.23 0.51 0.86
BMI [kg/m^2^]	23.8 ± 4.0	23.6 ± 3.9	23.8 ± 4.3	0.58	24.4 ± 4.0	24.4 ± 4.1	24.1 ± 3.6	
Familiarity [%]	31.9	30.3	34.1	0.62	43.2	41.0	53.8	
Age at menopause [years]	49.2 ± 4.7	49.1 ± 4.9	49.4 ± 4.6	0.47	49.2 ± 5.7	49.2 ± 5.8	49.2 ± 5.5	
Type of menopause [%]								
Early menopause	16.9	17.8	15.6	0.87 *0.025* 0.96 0.25	17.0	15.7	23.1	0.74
Physiological menopause	82.0	79.1	86.0		77.0	77.3	76.0	0.86
Surgery menopause	14.8	16.3	12.8		20.0	20.0	20.0	0.52
Pharmacological menopause	3.3	4.7	1.5		3.0	2.7	4.0	0.39
Breastfeeding [months]	8.9 ± 9.9	8.9 ± 10.1	8.8 ± 9.5	0.36	12.7 ± 14.6	12.9 ± 15.3	11.3 ± 10.4	0.89
Current smoking habits [%]	8.6	9.4	7.6	0.58	6.1	4.9	11.5	0.41
Previous smoking habits [%]	18.6	17.7	19.9	0.96	17.6	13.1	38.5	*0.002*
Years smoking	23.6 ± 13.8	24.0 ± 13.9	23.1 ± 13.7	0.63	31.5 ± 15.5	33.6 ± 17.3	27.2 ± 10.6	0.52
Susceptibility to falling [%]	5.6	5.9	5.3	0.54	16.2	17.2	11.5	0.11
Diabetes [%]	2.8	2.5	3.3	0.53	8.1	7.4	11.5	0.24
Drugs [%]								
Cortisone	8.1	10.3	5.0	*0.009*	23.0	20.5	34.5	0.74
Aromatase inhibitors	0.8	0.5	1.3	0.87	0.0	0.0	0.0	0.15
Anticoagulants	1.4	0.7	2.3	0.89	3.4	3.3	3.8	0.13
DEXA *T* score								
Lumbar spine	− 2.4 ± 1.0	− 2.7 ± 1.0	− 2.1 ± 1.0	< *0.001*	− 2.7 ± 1.3	− 2.8 ± 1.2	− 1.9 ± 1.4	< *0.001*
Total hip	− 1.6 ± 0.8	− 1.7 ± 0.8	− 1.4 ± 0.8	< *0.001*	− 2.4 ± 0.8	− 2.4 ± 0.8	− 2.1 ± 0.7	0.12
Vertebral fractures [%]	24.2	33.5	11.6	< *0.001*	75.7	83.6	38.5	< *0.001*
Femur fractures [%]	1.8	2.7	0.7	*0.045*	10.1	9.0	15.4	0.23
FRAX								
Major fractures	9.3 ± 4.4	10.3 ± 4.4	7.9 ± 4.1	< *0.001*	30.0 ± 11.1	30.4 ± 11.0	27.8 ± 11.2	0.25

Statistically significant *p* values are in italics

Numbers are means ± SD, medians (interquartile range), or counts (%), as appropriate

**Fig. 1:**
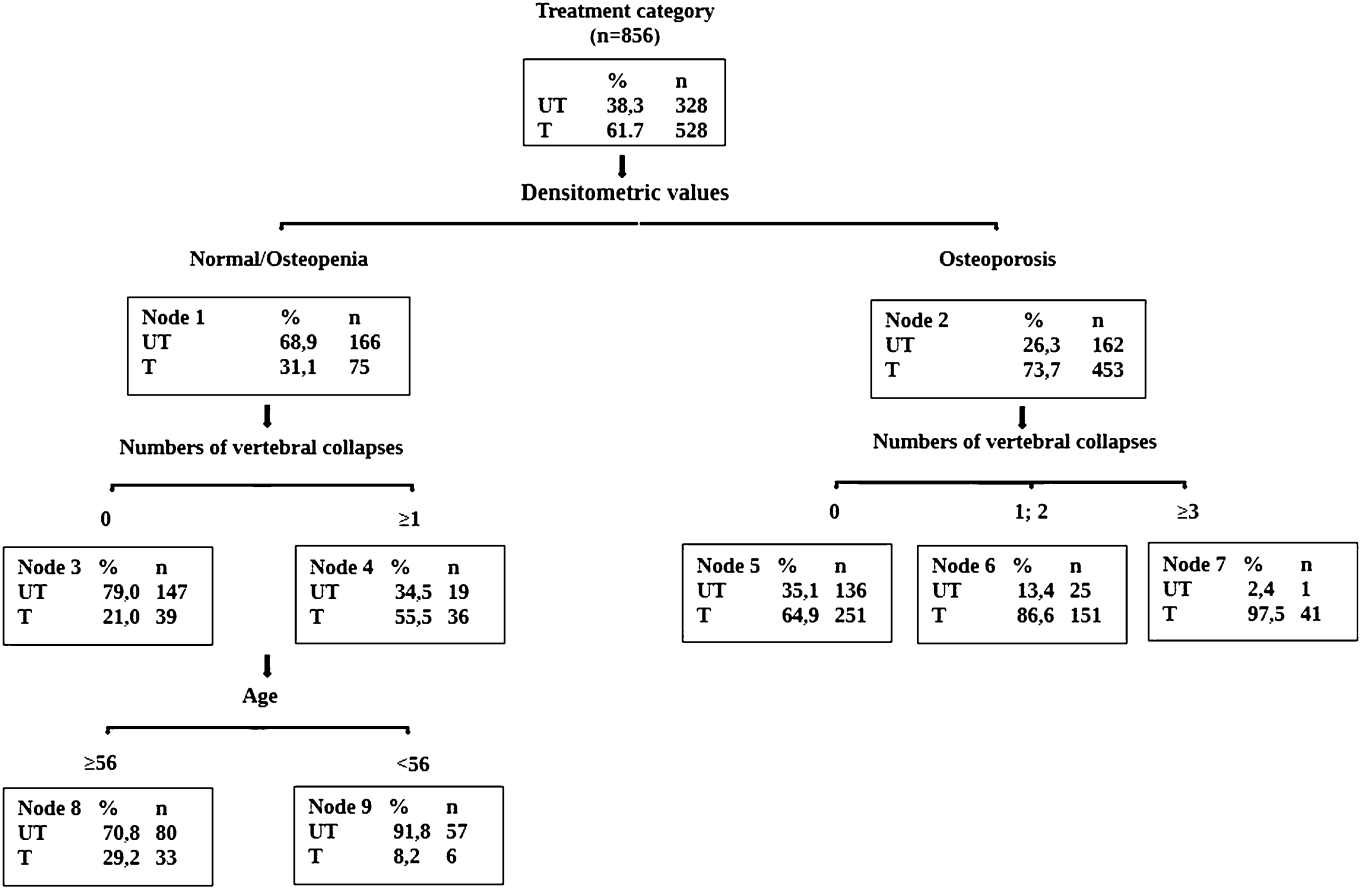
CHAID decision tree describing the decision-making process to determine outpatient therapy; *UT* untreated, *T* treated

The CHAID decision tree (Fig. 1) shows the factors that influenced the clinical decision to treat patients. The first choice was based on densitometric values. From this we treated 73.7% of patients with osteoporosis and 31.1% of osteopenic subjects. The second step was assessment of the number of vertebral fractures. Of the patients with osteoporosis, we treated about 65% who had no vertebral collapses, 86.6% with one or two vertebral collapses, and 97.6% with more than two vertebral collapses. We also treated 65.5% of osteopenic patients with 1 or more vertebral collapses. The risk estimate for the decision tree was 0.257, the standard error 0.015, which means that this classification tree analysis was able predict the decision to treat patients or not with an accuracy of approximately 74%. The decision tree had a sensitivity of 92.6% and a specificity of 44.8%; the positive predictive value was 73%, and the negative predictive value was 79%.

The follow-up period for all patients was 31.17 ± 22.78 months; for patients considered to be at low risk it was 28.93 ± 22.88 months (Table 3): in these subjects, we observed a greater loss of bone mineral density in the untreated group in both in the lumbar spine (0.16 ± 0.59 vs − 0.21 ± 0.49, *p* < 0.001) and the femur (0.07 ± 0.38 vs − 0.14 ± 0.40, *p* < 0.001).

**Table 3:** New vertebral fractures and *T* scores during the follow-up

FRAX < 20 (*n* = 708)			
Δ (visit 2–visit 1)	Treated (*n* = 406)	Untreated (*n* = 302)	*p *value
Δ T–s lumbar spine	0.16 ± 0.59	− 0.21 ± 0.49	< *0.001*
Δ T–s total hip	0.07 ± 0.38	− 0.14 ± 0.40	< *0.001*
Δ new vertebral fractures	9.9%	5.3%	*0.026*

Statistically significant *p* values are in italics

Δ = 31.17 ± 22.78 months

Regarding the onset of new major frailty fractures, the prevalence of vertebral fractures was higher in the treated patients compared to the untreated ones (5.3 vs 9.9%, *p* = 0.026), whereas we did not observe fractures in other skeletal sites.

## Discussion and conclusions

Our study shows that the clinical decision leads doctors to treat patients with osteoporosis who, based on the fracture risk calculated using the FRAX algorithm, should not be treated. The bone mineral density of both the femur and the spine seems to affect this choice. Conversely, a global clinical evaluation, which considers the needs and main problems of the elderly subject, and, therefore, the context in which the drug prescription takes place, would lead to the decision not to treat patients who, according to FRAX, would deserve preventive therapy.

Historically, treatment decisions for osteoporosis have been based on bone mineral density. However, many fractures occur in patients with *T* scores outside the osteoporotic range [8]. In light of this, the University of Sheffield developed the Fracture Risk Assessment tool (FRAX) to evaluate the 10-year probability of major osteoporotic fracture and hip fracture in men and women. The risks related to clinical variables (age, gender, body mass index, history of previous fractures, family history of fracture, smoking, alcohol use, rheumatoid arthritis, and glucocorticoid use) may be assessed by the FRAX algorithm alone or in combination with femoral neck BMD measured by DXA [16]. FRAX has been studied in different countries, and the tool should be calibrated on the basis of each country’s epidemiological data [16, 17].

FRAX is, therefore, a simple, objective tool for quantifying fracture risk. However, it only considers dichotomous data [9] and does not take into account a number of variables that are likely to have a dose–effect [18] (e.g., corticosteroid dose and duration, alcohol and tobacco consumption levels, activity and duration of predisposing diseases, such as rheumatoid arthritis and endocrinopathies), nor any drugs that potentially induce bone loss or increase the risk of fracture and risks associated with falls [4]. FRAX data, therefore, may not be sufficient for clinical decision making. A wider approach, such as the one taken in this study, could be useful for identifying patients at low or borderline risk, especially if they are older persons. The decision to treat these patients must take into account factors such as their ability to adhere to treatment, the possibility of correcting risk factors, the patient’s willingness to be treated, the family context, the cost–benefit ratio, and polypharmacy.

The decision tree shows that in the clinical outpatient evaluation the main factors guiding the doctor in the therapeutic decision making were the densitometric values and the presence of vertebral fractures. Our interest for vertebral collapses derived from their role on instability of the centre of gravity, and consequently disability. Furthermore, the deformity of the rib cage following vertebral collapse can compromise the respiratory dynamics, worsening the pictures of respiratory insufficiency which can be chronic [19, 20]. It, therefore, becomes essential in the elderly patient to prevent these complications, especially considering that vertebral fractures can also be completely asymptomatic.

At the same time, a wider assessment (typical of the geriatric approach) was carried out, which resulted in almost all the patients who FRAX had also identified as “at high risk of fracture” being selected for treatment, in line with the most recent guidelines [3].

However, our assessment also led us to treating patients whose FRAX scores identified them as “low fracture risk”, and, therefore, not necessitating therapy. Nonetheless, we treated 57% of these patients, who were older persons, more often in cortisone therapy or with pharmacologically induced menopause, and with lower densitometric values and a higher prevalence of osteoporotic fractures. Although the FRAX scores mean this therapeutic decision would be considered over-treatment, it was taken with the aim of preventing the onset of new fractures.

Hinz et al. [8] also found that clinical experience led doctors to overestimate the risk of fractures. In each proposed scenario, clinicians statistically significantly overestimated the fracture risk compared with the FRAX predicted probability. Nonetheless, their decision to offer drug treatment almost never differed from that suggested by FRAX scores and current guidelines. In this case, BMD was an important factor, followed by age, BMI, and smoking habit. When asked about the discriminating factors in “real life” treatment decision for medication, the main ones that emerged were prior fractures, BMD, age, BMI.

No femural fractures were observed in our sample maybe due to the young age of the patients.

In our study, at the control evaluation, we observed stable densitometric values at the femur and lumbar sites in the “low-risk-treated” group, and subsequently an unexpected increase in fractures: 9.9% presented at least one new vertebral fracture after 2 years. The reality was, therefore, much more serious than the predicted scenario, because fragility fractures occurred in a quarter of that time compared to what was predicted by the FRAX. The untreated group also exhibited a significant risk of fracture (5.3% of vertebral fractures in the investigation period vs an estimated FRAX risk of 7.9%). It is, therefore, clear that our approach selected a group of patients with a high fracture risk who had not been identified by FRAX, but were instead placed among the group of patients that did not require treatment.

Our results are surprising, especially given that the follow-up period was shorter than the average period reported in the literature. Indeed, the possibility that FRAX underestimate the risk of fracture is not new. A Canadian study [4] showed that FRAX underestimated the risk of fragility fracture, especially in menopausal patients, after a 4 year follow-up. A French study reported that the incidence of fractures within 10 years in women over the age of 65 with low BMD was significantly higher than predicted by FRAX [21]. In Switzerland, half of the patients analyzed were classified by FRAX as low risk the day before developing a fragility fracture [22]. According to Crandall et al. [23], the ability of FRAX to discriminate between women who will or will not experience a major osteoporotic fracture is no better than chance for postmenopausal women aged 50–64 [24, 25], and aged ≥ 65 years [26].

FRAX assesses the 10-year risk of fragility fractures in men and women over the age of 40 [27]. The major limit of this study is that our sample was not a random population, but comprised subjects who had been specifically referred to an outpatient clinic for the management of osteoporosis by a treating physician, or a gynecologist or oncologist specialist.

Conclusions. Although effective in general identification of subjects at high risk of fracture, assessment by the FRAX algorithm appears to underestimate the risk in older people. In these subjects, diagnostic-therapeutic decision making in real-world practice must consider a wider assessment focused on the individual patient and his/her needs.

## References

[1] Kanis JA, McCloskey EV, Johansson H, Oden A, Melton LJ, Khaltaev N (2008). A reference standard for the description of osteoporosis. Bone.

[2] Anastasilakis AD, Makras P (2020). Fracture risk among treatment-naïve postmenopausal women with osteopenia in Greece: results from the ‘ACROSS’ study. Arch Osteoporos.

[3] Kanis JA, Cooper C, Rizzoli R, Reginster JY (2019). European guidance for the diagnosis and management of osteoporosis in postmenopausal women. Osteoporos Int.

[4] Roux S (2014). The world health organization fracture risk assessment tool (FRAX) underestimates incident and recurrent fractures in consecutive patients with fragility fractures. J Clin Endocrinol Metab.

[5] Johansson H (2017). FRAX- vs. T-score-based intervention thresholds for osteoporosis. Osteoporos Int.

[6] Marshall D, Johnell O, Wedel H (1996). Meta-analysis of how well measures of bone mineral density predict occurrence of osteoporotic fractures. Br Med J.

[7] Johnell O (2005). Predictive value of BMD for hip and other fractures. J Bone Miner Res.

[8] Hinz L, Freiheit E, Kline G (2016). How good is our best guess? Clinical application of the WHO FRAX tool in osteoporotic fracture risk determination and treatment decisions. Calcif Tissue Int.

[9] Kanis JA (2020). A decade of FRAX: how has it changed the management of osteoporosis?. Aging Clin Exp Res.

[10] Kanis JA (2016). A systematic review of intervention thresholds based on FRAX: a report prepared for the national osteoporosis guideline group and the international osteoporosis foundation. Arch Osteoporos.

[11] Kanis JA, McCloskey EV, Harvey NC, Johansson H, Leslie WD (2015). Intervention thresholds and the diagnosis of osteoporosis. J Bone Miner Res.

[12] Genant HK, Wu CY, van Kuijk C, Nevitt MC (1993). Vertebral fracture assessment using a semiquantitative technique. J Bone Miner Res.

[13] Kass GV (1980) An Exploratory Technique for Investigating Large Quantities of Categorical Data. J Royal Stat Soc Series C (Applied Statistics) 29:119–127. 10.2307/2986296

[14] Song YY, Lu Y (2015). Decision tree methods: applications for classification and prediction. Shanghai Arch Psychiatry.

[15] IBM Corp (2012) “IBM SPSS decision trees 21.0”. Available: http://library.uvm.edu/services/statistics/SPSS21Manuals/IBMSPSSDecisionTrees.pdf. 118

[16] Oka R (2018). Fracture risk assessment tool (FRAX) and for the diagnosis of osteoporosis in Japanese middle-aged and elderly women: Chiba bone survey. Endocr J.

[17] Wang J, Wang X, Fang Z, Lu N, Han L (2017). The effect of FRAX on the prediction of osteoporotic fractures in urban middle-aged and elderly healthy Chinese adults. Clinics.

[18] Holloway-Kew KL (2019). How well do the FRAX (Australia) and Garvan calculators predict incident fractures? Data from the Geelong Osteoporosis Study. Osteoporos Int.

[19] Culham EG, Jimenez HAI, King CE (1994). Thoracic kyphosis, rib mobility, and lung volumes in normal women and women with osteoporosis. Spine (Phila. Pa. 1976).

[20] Harrison RA, Siminoski K, Vethanayagam D, Majumdar SR (2007). Osteoporosis-related kyphosis and impairments in pulmonary function: a systematic review. J Bone Miner Res.

[21] Sornay-Rendu E, Munoz F, Delmas PD, Chapurlat RD (2010). The FRAX tool in French women: how well does it describe the real incidence of fracture in the OFELY cohort. J Bone Miner Res.

[22] Aubry-Rozier B, Stoll D, Krieg MA, Lamy O, Hans D (2013). What was your fracture risk evaluated by FRAX® the day before your osteoporotic fracture?. Clin Rheumatol.

[23] Crandall CJ (2019). Do additional clinical risk factors improve the performance of fracture risk assessment tool (FRAX) among postmenopausal women? Findings from the women’s health initiative observational study and clinical trials. JBMR Plus.

[24] Crandall CJ (2014). Comparison of fracture risk prediction by the US preventive services task force strategy and two alternative strategies in women 50–64 years old in the women’s health initiative. J Clin Endocrinol Metab.

[25] Crandall CJ (2019). Predicting fracture risk in younger postmenopausal women: comparison of the Garvan and FRAX risk calculators in the women’s health initiative study. J Gen Intern Med.

[26] Ensrud KE (2009). A comparison of prediction models for fractures in older women: is more better?. Arch Intern Med.

[27] Chapurlat R (2013). Intérêt et limites du FRAX. Rev du Rhum.

